# The Healthy Hawai‘i Initiative: insights from two decades of building a culture of health in a multicultural state

**DOI:** 10.1186/s12889-019-8078-1

**Published:** 2020-01-31

**Authors:** Joy Agner, Catherine M. Pirkle, Lola Irvin, Jay E. Maddock, Opal Vanessa Buchthal, Jessica Yamauchi, Ranjani Starr, Tetine Sentell

**Affiliations:** 1 Department of Community and Cultural Psychology, University of Hawai‘i at Mānoa, 2530 Dole St, Honolulu, HI 96822 USA; 2 Office of Public Health Studies, University of Hawaii at Manoa, 1960 East West Road, Honolulu, HI 96822 USA; 3 Hawai‘i Department of Health, 1250 Punchbowl St, Honolulu, HI 96813 USA; 4 School of Public Health, Texas A&M University, 319 Administration Building, College Station, TX 77843 USA; 5 Office of Public Health Studies, University of Hawai‘i at Mānoa, 1960 East West Road, Honolulu, HI 96822 USA; 6 Hawai‘i Public Health Institute, 850 Richards St, Honolulu, HI 96813 USA; 7 Department of Health and Human Services, 1390 Miller St, Honolulu, HI 96813 USA

**Keywords:** Culture of health, Cross-sector, Health promotion, Hawaii, Built environment, Nutrition, Physical activity, Obesity

## Abstract

**Background:**

The Healthy Hawai‘i Initiative was created in 2000 with tobacco settlement funds as a theory-based statewide effort to promote health-supporting environments through systems and policy change. Still active today, it is imbedded explicitly in a multi-sectoral, social ecological approach, effectively striving to build a culture of health before this was the name for such an ambitious effort.

**Methods:**

From interviews with key informants, we analyze two decades of the Healthy Hawai‘i Initiative (HHI) in the context of the Robert Wood Johnson Foundation (RWJF) Culture of Health Action Framework (CHAF). We list HHI accomplishments and examine how the Initiative achieved notable policy and environmental changes supportive of population health.

**Results:**

The Healthy Hawai‘i Initiative started with an elaborate concept-mapping process that resulted in a common vision about making “the healthy choice the easiest choice.” Early on, the Initiative recognized that making health a shared value beyond the initial stakeholders required coalition and capacity building across a broad range of governmental and nonprofit actors. HHI coalitions were designed to promote grassroots mobilization and to link community leaders across sectors, and at their height, included over 500 members across all main islands of the state. Coalitions were particularly important for mobilizing rural communities. Additionally, the Initiative emphasized accessibility to public health data, published research, and evaluation reports, which strengthened the engagement to meet the shared vision and goals between diverse sector partners and HHI. Over the past two decades, HHI has capitalized on relationship building, data sharing, and storytelling to encourage a shared value of health among lawmakers, efforts which are believed to have led to the development of health policy champions. All of these factors combined, which centered on developing health as a shared value, have been fundamental to the success of the other three action areas of the CHAF over time.

**Conclusions:**

This evidence can provide critical insights for other communities at earlier stages of implementing broad, diverse, multifaceted system change and fills a key evidence gap around building a culture of health from a mature program in a notably multicultural state.

## Background

### A culture of health

Systematic investment in the prevention of chronic diseases is critical for healthy communities and for containing health systems costs [1–4]. As our understanding of chronic disease prevention improves, it is increasingly clear that a combined lifecourse and social ecological approach is necessary to improve community health [5, 6]. In 2016, the Robert Wood Johnson Foundation (RWJF) furthered this effort by providing a practical model to guide systems change: the Culture of Health Action Framework (CHAF) [7–9]. The CHAF action areas include (1) building social cohesion by creating health as a shared value, (2) cross-sector collaboration, (3) creating healthier, more equitable communities, and (4) strengthening integration of health services and systems [8, 9]. The CHAF builds on previous frameworks, models and efforts, including the Health-in-All policies and Healthy People 2020 [10]. A variety of far-reaching new programs have been explicitly designed under the CHAF, [11–13] and the literature on the process and outcomes of these programs is growing [14, 15].

However, critical research gaps remain. Given the recent development of the CHAF, most literature on this topic describes relatively new programs and only addresses one action area. This limits understanding of how the four action areas in the framework intersect, build on one another, and contribute to health policy and systems change in relationship to one another [8, 9, 11, 12, 14, 15]. Furthermore, limited work has considered building a culture of health in a Pacific context and/or included Native Hawaiians or heterogeneous Asian American populations [16–18].

Understanding of the complexity, broad scope, and scale needed to truly change multicultural communities towards health is limited. To reduce this knowledge gap, we offer a rich history of a mature, statewide program in a highly multicultural state according to the CHAF model. Specifically, we describe challenges and successful strategies around building a Culture of Health in Hawai‘i, analyze how the CHAF action areas were actualized over nearly 20 years through the Healthy Hawai‘i Initiative (HHI), and identify how the action areas have built on one another, resulting in progressive policy and built environment change. This can provide critical insights for other communities at earlier stages of implementing large-scale, long-term health promotion initiatives.

### Hawai‘i context

Hawai‘i is comprised of six main inhabited islands with strong urban/rural divides. The majority of the state’s population resides in Honolulu, and the state of Hawai‘i is extremely ethnically diverse [19]. Hawai‘i has strong health outcomes compared to the rest of the US, ranking low on obesity and tobacco use, with elevated relative longevity and high quality of life [19, 20]. However, Hawai‘i data shows an increase in chronic diseases and obesity over time [20]. The state has notable health disparities; compared to Whites, Native Hawaiians, Filipinos, and other Pacific Islanders are diagnosed with diabetes at earlier ages, have higher rates of hospitalizations for preventable chronic conditions and shorter longevity [19, 21, 22]. While Hawai‘i’s official poverty rate is very low, when cost-of-living is factored in, in 2017 Hawai‘i had the 10th highest poverty rate of all states, [23] and rural populations and lower income communities in the state have similar poor outcomes [19, 24]. Unlike many other US locations, Hawai’i has one centralized Department of Health (DOH) with satellite district health offices, and one state school district with 15 complex areas.

### Healthy Hawai‘i Initiative history

In 1999, the state of Hawai‘i passed Act 304, landmark legislation that mandated the Hawai‘i DOH expend at least 25% of tobacco settlement special funds (TSSF) on disease prevention programs and promotion of healthy lifestyles. The TSSF are from the state’s share of the Master Settlement Agreement, which required cigarette manufacturers to pay a $206 billion settlement along with restrictions on the sale and marketing of cigarettes [25]. The DOH effort was formalized in 2000 as the “Healthy Hawai‘i Initiative,” which was a Special Project within the Hawai‘i DOH. In 2014, the HHI program was institutionalized within the Chronic Disease Prevention & Health Promotion Division. The HHI always included a funded, active evaluation arm in partnership with the University of Hawai‘i’s Office of Public Health Studies, a best-practices approach. This long-lasting partnership has allowed rigorous evaluation across topic domains and a robust set of publications indicating its successes.

**Fig. 1:**
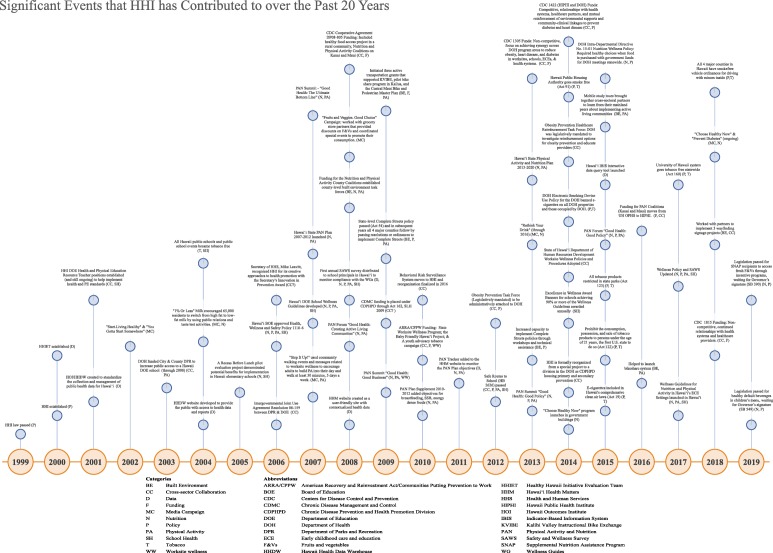
Timeline of major HHI activities and successes specifically focused on policy and environments change over the past 20 years

Initial HHI activities focused on meetings, trainings and workshops and grant-making to existing community programs to increase physical activity and accessibility of healthy food, as well as coordinated school health and social marketing initiatives [26, 27]. Over time, driven in part by Centers for Disease Control and Prevention (CDC) funding priorities, [28] programs have focused on multiple sectors with recent focus on collaborations with health systems [27]. A timeline of major HHI activities and successes specifically focused on policy and environments change can be seen in Fig. 1: *Significant Events that HHI has Contributed to Over the Past 20 Years*.

While this timeline gives a sense of *what* HHI has accomplished in the past 20 years, we utilize the CHAF to better understand *how* HHI achieved notable HHI policy and environment changes. Our objective is to analyze the HHI efforts, successes and challenges in order to inform current and future broad-scale initiatives seeking to build a culture of health. We chose the CHAF as a framework for this analysis because it is a highly influential model with clearly defined components that has in recent years become a critically important guide for designing, and also for funding, large-scale interventions. However, as this framework is quite new, little academic research has considered how the pieces fit together or been able to consider successes and challenges over the long time frame that cross-sector interventions may need to see meaningful changes in social norms and major policy. As the HHI enterprise has been in existence for 20 years, we felt there would be mutually beneficial learning for advancement of the evidence base of the CHAF and a more specific understanding of the working components of the CHAF.

## Methods

Ten in-depth, semi-structured interviews with key informants were conducted to better understand the history, achievements, and challenges of the HHI. Because of the massive scale of this 20-year initiative, key informants were purposefully selected to provide the greatest depth *and* breadth of knowledge on the HHI from a wide variety of perspectives (evaluation and research, program implementation, policy focus, school system focus, built environment, etc.), affiliations (DOH, non-profit, and university), and also time periods (from HHI initiation to current day). Table 1, *Key Informants,* provides further detail on their roles. Interview questions covered topics such as the initial vision and mission of HHI, strategies for HHI success, unique factors for implementing policy and environment changes within Hawai‘i, and essential steps to create a culture of health in the state. All participants provided informed consent. Interviews were audio-recorded. Interviews were transcribed by Rev.com and Temi and transcripts of these interviews were stored on password-protected computers available only to the research team. No participants are identified in dissemination materials.

**Table 1:** Key Informants

	Affiliation	Role
1	University of Hawai‘i at Mānoa	Former Healthy Hawai‘i Initiative Evaluation team member and Principal Investigator
2	University of Hawai‘i at Mānoa	Healthy Hawai‘i Initiative Evaluation team member
3	Hawai‘i Department of Heath	Senior employee at the Chronic Disease Prevention and Health Promotion Division
4	Hawai‘i Department of Heath	Epidemiologist at the Chronic Disease Prevention and Health Promotion Division
5	University of Hawai‘i at Mānoa	Health Hawai‘i Initiative Evaluation team member
6	University of Hawai‘i at Mānoa	Health Hawai‘i Initiative Evaluation team member
7	Hawai‘i Department of Heath	Senior employee at the Chronic Disease Prevention and Health Promotion Division
8	Hawai‘i Department of Heath	Senior employee at the Chronic Disease Prevention and Health Promotion Division
9	Non-Profit Stakeholder Partner	Executive Director of a Stakeholder Organization
10	Non-Profit Stakeholder Partner	Executive Director of a Stakeholder Organization

Transcripts were then analyzed in NVivo 11 both deductively and inductively by 2 authors (JA,TS). Qualitative analysis was completed using the framework method, which is useful for analyzing complex, multifaceted processes [29]. Prior to the interview process, the CHAF was chosen as an ideal framework because it is intended to guide large-scale, environment and policy change to create a culture of health. Deductive analysis utilized the four domains of the CHAF. Inductive analysis allowed unique sub-themes to emerge. Emergent subthemes were analyzed iteratively through a process of memoing and charting. Charting involves placing sub-themes into a matrix, based on the framework chosen for analysis. This encourages identification of relationships between themes, and for new insights on the analytical framework to emerge based on its application to a unique setting.

The interview analysis was complemented by a targeted document review. Research and evaluation was an ongoing aspect of the HHI initiative, which provided a rich resource of both academic publications and evaluation reports over 20 years. While a systematic literature review of HHI reports and articles is beyond the scope of this article, a selective review of HHI literature and evaluation reports was essential not only for triangulation of findings, but to add context and supporting details to key informant reflections. For example, after a theme emerged on the importance of coalitions for cross-sector collaboration, journal publications on HHI coalitions were reviewed, as well as details on coalition development within the evaluation reports. After a theme emerged on the importance of educating policymakers, publications and evaluation reports were reviewed concerning that topic specifically. Triangulation through document review added credibility and trustworthiness to the qualitative analysis. Quotes and details from these documents are included in the results sections, alongside themes and quotes from the key informants.

## Results

**Fig. 2:**
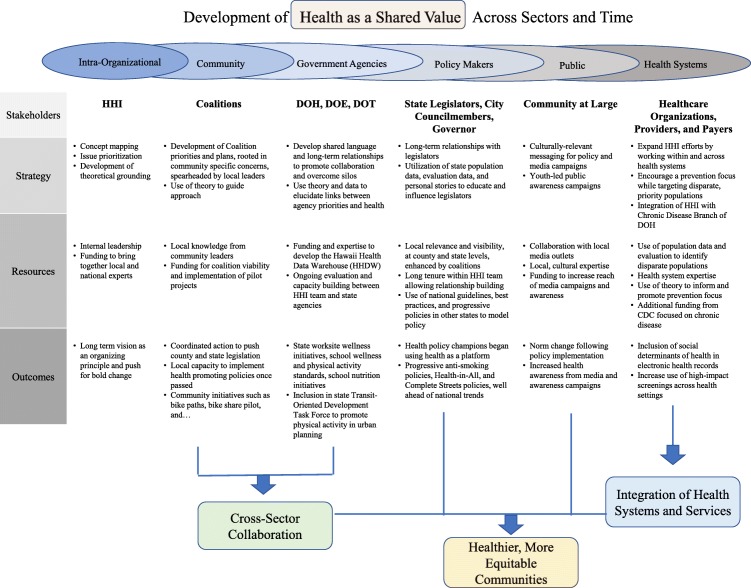
Development of health as a shared value across sectors and time

The purpose of this study was to retrospectively analyze HHI efforts and successes – which include policy adoption and implementation, systems change (within schools and healthcare), and built environment change, using the CHAF. We were particularly interested in how the HHI initiatives were accomplished, and how they contribute to a culture of health. Subthemes emerged in each action area from CHAF, and the action areas built on each other over time. An overview of this process and the results can be found in Fig. 2: *Development of Health as a Shared Value Across Sectors and Time*. Each of the action areas is introduced below, followed by insights how they contributed over time to community, policy, and environment change.

### Making health a shared value: starting internally with a clear, bold vision

We found that developing a shared vision among HHI stakeholders was a crucial first step to developing policies and environments supportive of statewide health. Several key informants described that a clearly specified long-term vision and theoretical foundation was essential to mobilize communities around a shared set of values and goals, to evaluate the success of the HHI initiative over time, and to maintain cohesion among programs within this long-term, large-scale undertaking.

HHI leaders developed a shared vision through an extensive concept mapping process involving the DOH, national experts, community leaders, and health scholars [14]. Participants created 448 health priority statements, and then rated and sorted them by feasibility and importance. This process resulted in a strategic plan to promote policy and environment changes focused on reducing three core behaviors that contribute significantly to chronic disease: smoking, inactivity and poor diet [14]. From this initial meeting onward, the vision of HHI was to build a culture of health with an explicit focus on making the healthy choice the easiest choice, one that is prompted and promoted by the environment.

While the concept mapping process aided in developing a shared vision among HHI stakeholders, *how* that vison would be realized was informed by CDC best practices [4] and social ecological theory. The socio-ecological model informed program development, shaped evaluation questions and metrics, and encouraged community partners see how policy, system, and environment affected community health [30]. Cross-sector collaboration was used [31, 32, 33, 34]. In this, communty partners were encouraged to use the socio-ecological model, and to focus on the outer rings of it. Using language that was easy to understand, making theory simple and approachable, was key to successful collaboration with community partners from a variety of backgrounds.

The bold focus on policy and environment change, and the use of theory and science was not always readily embraced, particularly in institutional settings. A key informant from the DOH described, “*There is just this natural tension between this very ambitious group that HHI was and… programs that weren’t indoctrinated into that culture to the same extent*.” Institutional cultural differences made policy implementation a challenge, and the cultural and geographic diversity of Hawai’i made it difficult to develop a vision that was both population-based *and* targeted to priority populations. It became clear early on that realizing the bold vision of HHI required making health a shared value beyond the initial HHI stakeholders, and required coalition and capacity building across a broad range of governmental and nonprofit actors.

### Developing cross-sector collaboration through coalitions and capacity building

Cross-sector collaboration was part of HHI’s initial long-term vision. HHI guiding principles stated that an “integrated, non-categorical approach” across sectors and “authentic community ownership” were essential to system, environment, and policy changes to prevent chronic disease [35]. A key strategy to achieve these aims was the development and support of coalitions. HHI coalitions were designed to promote grassroots mobilization and to link community leaders across sectors. At their height, HHI associated coalitions included over 500 members across five Hawaiian islands [26]. They were action-oriented, and organized around topics such as preventing childhood obesity, reducing smoking rates, and developing infrastructure for active commuting [26, 36]. They implemented a wide range of initiatives from reclaiming abandoned airports to build multi-use recreational paths, to mobilizing youth to resist cigarette advertising, to legislative advocacy [35, 37]. Key informants described coalitions as essential for developing locally-relevant ideas while advancing the HHI vision and incorporating CDC best practices. Coalitions were seen as particularly important outside of O‘ahu, where it was more difficult for HHI staff to engage local communities due to geographic barriers.

Coalition initiation, sustainability and success required consistent funding, direction, and early evidence of small wins. Several key informants described the importance of committed and charismatic coalition leadership. The ability to engage coalition members in efforts they saw as directly influential to their communities was essential. Detriments to coalition success included inconsistent funding, changes in coalition leadership, burdensome bureaucratic process, and restricting coalition agency by asking members to adopt initiatives viewed as irrelevant to their communities. Although government funding was a crucial support, several informants described tension negotiating government regulation and bureaucracy with coalition viability and flexibility.

Developing trust and shared language among stakeholders was also crucial to coalition success, which is well substantiated in collaboration literature [38, 39]. There was significant time spent bringing diverse stakeholders together for the sole purpose of developing relationships. Over time, coalition networks proved essential to passing and implementing comprehensive smoke-free, safe routes to school and complete streets legislation that involved multiple agencies [26, 36]. “*Now people at the Department of Transportation, people at the Department of Public Works, Departments of Planning ... they all know that the Department of Health is interested in healthy community design, and that they have resources that they can give to partners to help move the work along*.”

Funding was essential to coalition success and inter-agency collaboration, as was technical assistance: “*What HHI has done as a model is they’ve brought in national consultants and they’ve offered training and technical assistance to all the government departments that are responsible for actually making the built and policy environment*.” As coalitions and diverse agencies supported the implementation of health promotion initiatives, the HHI evaluation team developed metrics that demonstrated the impact of HHI efforts and helped identify areas for improvement. This prompted the need for relevant and accessible health surveillance data, particularly by disaggregated Native Hawaiian, Pacific Islander and different Asian American groups to identify needs relevant to the community [16].

The online resource, Hawai‘i Health Data Warehouse, was concurrently developed and improved under the culture of health idea that “*what gets measured is what gets changed*.” [16, 25] After the data platform was established, HHI trained community partners how to utilize surveillance data to answer their own performance related questions. One key informant described sharing evaluation tools with the Department of Education: “*While [teachers] may not be initially comfortable with data, when they’re able to run some of the IBIS [Indicator-Based Information System] data and find out exactly what they need, they get so excited to be able to use this*.” Empowering teachers to access data directly made the connections between health and academic achievements clear, and provided an opportunity to use health data directly for program improvement.

Surveillance data and research also helped bridge what seemed to be competing interests between agencies and demonstrate the interconnectedness of multiple outcomes, such as nutrition, education, and physical activity. “*The HHI team at DOH has really done well to help frame the health needs, and how they are actually going to benefit not just the students but the schools… Essentially, [they’re] finding out what the interests and goals are for the other agency or sector and…kind of spin it for them so they can find the data to be useful*.” Building cross-sector collaboration required making health a shared value in coalitions as well as government agencies. The intentional accessibility to public health data, published research, and evaluation reports strengthened the engagement to meet the shared vision and goals between diverse sector partners and HHI.

### Engaging legislators to build healthier, more equitable communities

Building healthier, more equitable communities required overcoming the difficult challenge of getting lawmakers' *“attention to prevention,”* and developing a shared perspective on *how* to build healthy communities, either through built environment changes or policy. To develop a shared vision with lawmakers, HHI utilized a combination of long-term relationship building, task forces, individual meetings, evidence, and personal stories that made the value of health policy apparent. “*One thing that we took from very early on was legislative education, and building up the knowledge based on what public health is and why policies make a difference.*” Meeting early and often with lawmakers was key to developing a shared vision of health, as lawmakers had often made up their mind on an issue by the time an opportunity came for public comment, and personal relationships made legislators more likely to listen [40].

Consistent with evidence-based policy-making, HHI used a combination of state surveillance data, opinion polls, and personal stories to make health a priority to lawmakers [40]. Several informants described a cultural value of children’s health, especially with regard to smoke-free legislation, which was used to encourage buy-in from lawmakers and reinforced by Project REAL, a youth led coalition fighting to reduce tobacco use. “*We always had the data, and we always presented with data. But…we knew that for some legislators if we brought in kids, and kids told their stories, that’s the evidence they wanted to hear.*” In addition to presenting compelling data with personal stories, it was important to demonstrate the relevance to individual districts. Legislators are not typically divided along party lines in Hawai‘i, but they do advocate strongly for their districts. It was, thus, essential to have coalitions in all of the counties to show that efforts were being made to promote health across the islands, in every community.

Decades of relationship building, data sharing, and storytelling encouraged the development of a shared value of health among lawmakers and led to health policy champions. In addition to tobacco prevention advocacy, legislators became involved in media campaigns around active transport and used health policy in their campaigns as a way to appeal to their constituents. HHI’s policy achievements primarily support two of the three goals of the initial HHI vision: tobacco prevention and changing the physical environment to promote physical activity. In the following section, we describe the dynamic interplay between policy, environment, and norm change, and present one of the areas that proved to be a significant political challenge.

### Encouraging health as a shared value within the broader community: Norm change and media campaigns

We found that norm change, and its dynamic interplay with policy environments, is key to making health a shared value in the broader community. Key informants described nutrition policy efforts, such as a sugary beverage fee, as politically unviable, partially because it was difficult to align food environment changes with local norms. Informants described that nutrition policy is often more nuanced than tobacco legislation, which made public messaging more difficult, and policies harder to pass. “*You don’t need any tobacco, and there’s no level of tobacco that’s good for you. So, it’s really black and white*.” Other advantages that made progressive tobacco prevention policies more viable include the Surgeon General’s report, model policy from other states, and report cards from the American Lung Association; parallel national standards and model policies were not available for many nutritional intitiatives.

However, in areas where nutrition-focused worksite or school policies were implemented, such as limiting sugary beverages in schools, norm change followed. Similarly, tobacco prevention legislation was not initially popular, but after policies were passed, smoking became less fashionable and acceptable. One informant described, “*I think there’s some chicken and egg with the shift in community norms [around smoking]. Some of the community norms have been driven by policy changes. And some of the policy changes were certainly driven by more people in the general public.*” Key informants described a synergy and an interplay between policies and behaviors that are essential to ingraining behaviors and mindsets that create a culture of health.

Media campaigns specifically sought norm change in food choice, such as “Rethink Your Drink” (to reduce sugary beverage consumption); “One Percent or Less” (to encourage consumption of low-fat milk); Start.Living.Healthy (promotion of small, manageable changes to diet and activity); and “Five-A-Day” (to promote eating more fruits and vegetables). The media campaigns reached a significant portion of the population, were described as *“a starting point for work with communities,”* and as one aspect of a multi-pronged approach to shift norms and behaviors. However, despite a culturally-tailored approach, they did not reach low-income audiences as effectively as middle/upper income groups [41]. Particularly among low-income groups, key informants described cost and peer influences as the crucial to dietary changes. “*If the healthy food is the cheaper food, and the food I see all around me, and the food that I see everyone eating, that’s what I’ll do*.”

### Health disparities persist: Next steps and integrating health systems and services

Thus far, we have addressed HHI efforts to create health as a shared value internally, with coalition members, across sectors, among legislators, and eventually the broader community, as well as the notable policy accomplishments and environment changes to promote health and well-being across the state. However, several informants described that the implementation of policy and environment changes takes time. “*It’s a really slow process, because not only do policies have to be in place, but then there’s the whole staff capacity building piece… But you can see in Kaua‘i now, a small example where it’s taken all these years to get their [Complete Streets] policies in in place, now we’re starting to see changes on the ground.*” However, many informants noted that even as population health improves, health disparities persist. When asked about challenges, one informant described, “*I don’t think we ever really made a huge difference in health disparities…certainly the SNAP Ed program did look for lower income folks. But the life expectancy [gap] between Hawaiians and Japanese has always been big and we didn’t shrink it.*”

Challenges associated with addressing health disparities included developing culturally targeted interventions in a multicultural state, and implementing built environment policies that applied equally well to urban and rural environments. In rural environments and more isolated communities, the socio-ecological model is not always as straightforward in application. Guidelines and models for developing walkable communities or transforming food deserts tend to focus on urban environments. Working at different levels of prevention in vastly different environments is necessary to address persistent disparities, and HHI is increasingly integrating prevention efforts with health systems and services to take on this challenge.

However, focusing on prevention in health systems and services environments demands a different set of skills and strategies than passing tobacco prevention legislation or developing walkable communities. It also requires engagement and relationship building with a new set of partners that operate in a parallel system to the DOH. Because the United States health system is fragmented, integrating prevention services with consistency is incredibly complex.

Furthermore, while HHI has taken a population health approach focused on the outer rings of the ecological model, medical systems tend to focus on individual treatments, which presents a challenge to establishing a shared vision with these partners. One key informant described, “*I have been fighting really hard to blend the culture of policy systems and environmental change into our chronic disease efforts, so I think the idea of how do you take the chronic disease strategies that we want to move, but do them at a level that will produce systems-level changes is a really difficult process.*”

Work to integrate health services and systems into HHI efforts sheds light on the fractured continuum of care between chronic disease prevention and treatment. HHI is currently exploring ways to create a more cohesive system, one that simultaneously maintains a view of chronic disease treatment and prevention. Current strategies underway include promoting community-clinical linkages and increasing incentives for high-impact screenings, particularly among priority populations. Changes in the for-profit healthcare system require a business-minded approach, such as financially incentivizing services that focus on prevention, detection, and health behaviors, and connecting prevention efforts to performance standards. Additionally, increasing alignment between federal funding agencies like Centers for Medicaid and Medicare Services and CDC would help integrate treatment and prevention efforts. Within the state, a shared vision of health between major health system players (Federally Qualified Health Centers, Kaiser, HMSA, Ohana Care, etc.) needs to be developed so that they are offering similar services with the goal of prevention and early, effective treatment.

Development of a shared vision and more integrated prevention and treatment services also requires increased connectivity between systems. We need to find ways that community organizations, public health experts, and health systems can share strategies, knowledge, and information to collaborate on interventions to reach priority populations. Within health systems, this can include increasing compatibility of electronic health records so patients have an improved continuum of care, and linking leaders of diverse agencies. Finally, we need to develop a shared vision of how to improve quality of care and build trust with disenfranchised communities. Often priority populations, or those that are the most vulnerable with the highest health needs, rate their healthcare experiences as worse than the general population [42]. Trust, respect, access, and information are key elements of effective care, engaging hard-to-reach populations, and linking HHI’s prevention focus with reducing health disparities.

## Discussion

Findings from the HHI experience over 20 years also add new insights to the Culture of Health literature, particularly the synergy and timing between the action areas, and the critical formative importance of developing health as a shared value. Making health a shared value requires a cultural shift, where health is prioritized as a value across sectors and among the general public [29]. We found that developing health as a shared value began within the organization with internal goals and visioning process. This laid the foundation for work with community partners and coalition building, which extended into a shared vision of health with legislative champions, and finally the broader public. At all levels it involves a dynamic interplay between systems change and norm change. While this differs from the original conceptualization of making health a shared value, [29] we believe it contributes a new understanding which can be useful to long-term public health initiatives.

Our findings also build upon past research on cross-sector collaboration, which are instrumental in reducing preventable deaths attributable to chronic disease [31, 32]. Ameliorating health inequities requires a multisystemic approach [33, 34]. We found that in HHI cross-sector collaboration was largely accomplished through coalition work. Our findings align closely with a large body of research on intersectoral collaboration that illustrates the importance of trust building, developing shared language and a shared vision among diverse stakeholders, supporting champions of that vision, and celebrating small wins along the way [31, 38, 39, 46]. Increasing access to surveillance data, and capacity to understand those data and their application, furthered collaboration by illustrating how multiple systems and interventions were interrelated (e.g. connections between academic achievement and exercise or nutrition).

In addition to offering new analysis of the CHAF, our findings contribute to multiple bodies of literature within health policy and public health. This work is congruent with research that shows framing prevention efforts around “vulnerable” populations, especially children, and combining stories with data, are successful strategies to convince policy makers [43–45]. Furthermore, our findings build upon literature that highlights the tensions and challenges inherent in building healthier, more equitable communities. Hawai‘i has made significant reductions in smoking among both youth and adults; however, electronic cigarette use is rising. In counties where Complete Streets and Safe Routes to School have been passed and implemented with the support of coalitions, (such as Kaua‘i﻿) physical activity rates are increasing. HHI successfully partnered with the DOT and DOE School Food Services to provide fresh, locally grown food in public schools. Yet statewide, obesity is rising, and the state has not yet established a sugary beverage fee, despite consistent advocacy for this goal since 2010 and important evidence that such a fee could reduce obesity rates [47]. These contradictions demonstrate the complexity of shifting health outcomes, even with decades of concentrated, organized efforts.

We also note practical challenges between building healthier communities and building more equitable communities, while recognizing that both are important and work synergistically [44]. A population approach that shifts the mean distribution of risk factors can accentuate disparities, even if population outcomes improve as a whole [44]. Recent CDC grants include an explicit focus on health disparities and vulnerable populations, which stems from Healthy People 2020 goals. The focus on population-based health outcomes and achieving health equity requires different strategies and resource allocations than those targeting population health, and there can be a tension between activities suggested by the traditional focus on shifting means for the whole state versus reducing health disparities. This is something the state of Hawai‘i, along with many locations building a widespread culture of health, must consider.

## Conclusions

Looking back on a major, statewide initiative across almost 20 years of chronic disease prevention in Hawai’i provides insights on how long-term, large-scale public efforts can promote health policy and environment change. We used the RWJ Culture of Health Action Framework to better understand 20 years of health policy and environment change efforts in Hawaii. The CHAF is typically used to inform program *development*, so part of the original contribution of this work is to see whether it is also theoretically useful to retrospectively analyse a long-term, state-wide process of change. We found that it was, and that each of the domains built on each other over time, with health as a shared value fundamentally underpinning each domain. This work also provides insights on next steps for the HHI, which will continue a population-based prevention focus, while utilizing integrated data systems, culturally-based approaches, and community-clinical linkages to target persistent disparities among Native Hawaiian, Pacific Islander, and Asian American populations [16, 48].

## Data Availability

The datasets analyzed during the current study are available from the corresponding author on reasonable request.

## Abbreviations

CDC: Centers for Disease Control and Prevention
CHAF: Culture of Health Action framework
DOH: Department of Health
HHI: Healthy Hawai‘i Initiative
RWJF: Robert Wood Johnson Foundation
TSSF: Tobacco settlement special funds

## References

[1] World Health Organization (2005). WHO | Preventing chronic diseases: a vital investment.

[2] Trust for America’s Health (2009). Prevention for a Healthier America: Investments in Disease Prevention Yield Significant Savings, Stronger Communities.

[3] Park BZ. State Public Health Actions to Prevent and Control Diabetes, Heart Disease, Obesity and Associated Risk Factors, and Promote School Health. Prev Chronic Dis [Internet]. 2017 [cited 2019 Apr 1];14. Available from: https://www.cdc.gov/pcd/Issues/2017/16_0437.htm 10.5888/pcd14.160437 PMC5724997 29215978

[4] Rutledge GE, Lane K, Merlo C, Elmi J (2018). Coordinated Approaches to Strengthen State and Local Public Health Actions to Prevent Obesity, Diabetes, and Heart Disease and Stroke. Prev Chronic Dis.

[5] Stokols D (1996). Translating social ecological theory into guidelines for community health promotion. Am J Health Promot.

[6] Lynch J, Smith GD (2005). A life course approach to chronic disease epidemiology. Annu Rev Public Health.

[7] Weil AR, Building A (2016). Culture of health. Health Aff.

[8] Weil AR (2016). Defining and measuring a culture of health. Health Aff.

[9] Trujillo MD, Plough A (2016). Building a culture of health: a new framework and measures for health and health care in America. Soc Sci Med.

[10] Rudolph L, Caplan J. Health in All Policies: A Guide for State and Local Government [Internet]. Public Health Institute; 2013 [cited 2019 Apr 1]. Available from: http://www.phi.org/resources/?resource=hiapguide

[11] Hiatt RA, Sibley A, Fejerman L, Glantz S, Nguyen T, Pasick R (2018). The San Francisco Cancer initiative: a community effort to reduce the population burden of Cancer. Health Aff.

[12] Acosta JD, Whitley MD, May LW, Dubowitz T, Williams MV, Chandra A. Stakeholder Perspectives on a Culture of Health. Rand Health Q. 2017;6(3) [cited 2019 Apr 1]. Available from: https://www.ncbi.nlm.nih.gov/pmc/articles/PMC5568151/. 10.7249/rhq-06-03-06 PMC5568151 28845358

[13] Robert Wood Johnson Foundation. Sentinel Communities - RWJF [Internet]. Sentinel communities: How communities work towards health. 2016 [cited 2019 Apr 1]. Available from: https://www.rwjf.org/en/cultureofhealth/what-were-learning/sentinel-communities.html

[14] Trochim WMK, Milstein B, Wood BJ, Jackson S, Pressler V (2004). Setting objectives for community and systems change: an application of concept mapping for planning a statewide health improvement initiative. Health Promot Pract.

[15] Madison KM (2016). The risks of using workplace wellness programs to foster a culture of health. Health Aff.

[16] Rubin V, Ngo D, Ross A, Butler D, Nisha B (2018). Counting a diverse nation: disaggregating data on race and ethnicity to advance a culture of health.

[17] Chung-Do JJ, Look MA, Mabellos T, Trask-Batti M, Burke K, Mau MKLM (2016). Engaging Pacific islanders in research: community recommendations. Prog Community Health Partnersh.

[18] Chung-Do JJ, Davis E, Lee S, Jokura Y, Choy L, Maddock JE (2011). An observational study of physical activity in parks in Asian and Pacific islander communities in urban Honolulu, Hawaii, 2009. Prev Chronic Dis.

[19] Wu Y, Braun K, Onaka AT, Horiuchi BY, Tottori CJ, Wilkens L (2017). Life expectancies in Hawai‘i: a multi-ethnic analysis of 2010 life tables. Hawaii J Med Public Health.

[20] Hawaii State Department of Health. Hawaii Coordinated Chronic Disease Framework [Internet]. Chronic Disease and Health Promotion Division; 2014 [cited 2019 Apr 1]. Available from: http://health.hawaii.gov/chronic-disease/files/2014/09/CDFrameworkLR.pdf

[21] Sentell TL, Seto TB, Young MM, Vawer M, Quensell ML, Braun KL (2016). Pathways to potentially preventable hospitalizations for diabetes and heart failure: a qualitative analysis of patient perspectives. BMC Health Serv Res.

[22] Look MA, Trask-Batti MK, Agres R, Mau ML, Kaholokula JK (2013). Assessment and priorities for health & well-being in Native Hawaiians & other Pacific Peoples. Center for Native and Pacific Islander Research.

[23] US Census Bureau (2017). The Supplemental Poverty Measure: 2017.

[24] Holmes JR, Tootoo JL, Chosy EJ, Bowie AY, Starr RR (2018). Examining Variation in Life Expectancy Estimates by ZIP Code Tabulation Area (ZCTA) in Hawaii’s Four Main Counties, 2008–2012. Prev Chronic Dis.

[25] Irvin LH, Johnson L, Yamauchi J, Holmes JR, Ching LK, Starr RR (2019). Formative factors for a statewide tobacco control advocacy infrastructure: insights from Hawai‘i. Hawaii J Med Public Health.

[26] Maddock JE, Aki NN, Irvin LH, Dang JF (2007). Using coalitions to address childhood obesity: the Hawai‘i nutrition and physical activity coalition. Hawaii Med J.

[27] Buchthal OV, Maddock JE (2015). Mapping the Possibilities: Using Network Analysis to Identify Opportunities for Building Nutrition Partnerships Within Diverse Low-Income Communities. J Nutr Educ Behav.

[28] Matsuoka CT, Nett B, Stromberg H, Maddock JE (2005). Improving access to physical activity: revitalizing the old Kona airport walking/jogging path. Californian J Health Promot.

[29] Gale NK, Heath G, Cameron E, Rashid S, Redwood S. Using the framework method for the analysis of qualitative data in multi-disciplinary health research. BMC Med Res Methodol. 2013;13(1). 10.1186/1471-2288-13-117. 10.1186/1471-2288-13-117 PMC3848812 24047204

[30] Nigg C, Maddock J, Yamauchi J, Pressler V, Wood B, Jackson S (2005). The healthy Hawaii initiative: a social ecological approach promoting healthy communities. Am J Health Promot.

[31] Hogg RA, Varda D (2016). Insights into collaborative networks of nonprofit, private, and public organizations that address complex health issues. Health Aff.

[32] Mays GP, Mamaril CB, Timsina LR (2016). Preventable death rates fell where communities expanded population health activities through multisector networks. Health Aff.

[33] Furtado KS, Brownson C, Fershteyn Z, Macchi M, Eyler A, Valko C (2018). Health departments with a commitment to health equity: a more skilled workforce and higher-quality collaborations. Health Aff.

[34] Siegel B, Erickson J, Milstein B, Pritchard KE (2018). Multisector partnerships need further development to fulfill aspirations for transforming regional health and well-being. Health Aff.

[35] Hawai’i Department of Health (2004). State of Hawai’i Department of Health Report to the 2004 Legislature Pursuant to the Act 200, Session Laws of Hawi’i 2003.

[36] Heinrich KM, Aki NN, Hansen-Smith H, Fenton M, Maddock J (2011). A comprehensive multi-level approach for passing safe routes to school and complete streets policies in Hawaii. J Phys Act Health.

[37] Choy LB, Maddock JE, Brody B, Richards KL, Braun KL (2016). Examining the role of a community coalition in facilitating policy and environmental changes to promote physical activity: the case of get fit Kaua‘i. Transl Behav Med.

[38] Bryson JM, Crosby BC, Stone MM (2015). Designing and implementing cross-sector collaborations: needed and challenging. Public Adm Rev.

[39] Vangen S, Huxham C (2003). Nurturing collaborative relations Building trust in interorganizational collaboration. J Appl Behav Sci.

[40] Brownson RC, Chriqui JF, Stamatakis KA (2009). Understanding evidence-based public health policy. Am J Public Health.

[41] Buchthal OV, Doff AL, Hsu LA, Silbanuz A, Heinrich KM, Maddock JE (2011). Avoiding a knowledge gap in a multiethnic statewide social marketing campaign: is cultural tailoring sufficient?. J Health Commun.

[42] Agency for Healthcare Quality and Research (2017). 2017 National Healthcare Quality and Disparities Report.

[43] Oliver K, Innvar S, Lorenc T, Woodman J, Thomas J. A systematic review of barriers to and facilitators of the use of evidence by policymakers. BMC Health Serv Res. 2014;14(1) [cited 2019 Apr 1];. Available from: https://bmchealthservres.biomedcentral.com/articles/10.1186/1472-6963-14-2. 10.1186/1472-6963-14-2 PMC3909454 24383766

[44] Frohlich KL, Potvin L (2008). Transcending the known in public health practice: the inequality paradox: the population approach and vulnerable populations. Am J Public Health.

[45] Brown LD (2010). The political face of public health. Public Health Rev.

[46] Ansell C, Gash A (2007). Collaborative governance in theory and practice. J Public Adm Res Theory.

[47] Choy L, Dela Cruz MR, Hagiwara M, Hee Heo H, Peacock T, Pearce MG (2013). Taxing sugar sweetened beverages to improve public health: policy action in Hawai‘i. Hawaii J Med Public Health.

[48] Chosy J, Benson K, Belen D, Starr R, Lowery St John T, Starr RR (2015). For the love of data! The Hawai‘i health data warehouse. Hawaii J Med Public Health.

